# Distinct memory CD4^+^ T cell subset tropism of two CCR5-tropic HIV-1 in a rapid progressor

**DOI:** 10.1128/asmcr.00101-25

**Published:** 2025-08-26

**Authors:** Manukumar Honnayakanahalli Marichannegowda, Yasmine Farah, Meera Bose, Eric Sanders-Buell, David King, Leilani Francisco, Leigh Anne Eller, Abdur Rashid, Sodsai Tovanabutra, Nelson L. Michael, Merlin L. Robb, Hongshuo Song

**Affiliations:** 1 Institute of Human Virology, University of Maryland School of Medicine 12264, Baltimore, Maryland, USA; 2 U.S. Military HIV Research Program, Walter Reed Army Institute of Research 8394, Silver Spring, Maryland, USA; 3 The Henry M. Jackson Foundation for the Advancement of Military Medicine, Inc., Bethesda, Maryland, USA; 4 Morsani College of Medicine, University of South Florida 33697, Tampa, Florida, USA; 5 Center for Infectious Diseases Research, Walter Reed Army Institute of Research 8394, Silver Spring, Maryland, USA; 6 Global Virus Network, Tampa, Florida, USA; Rush University Medical Center, Chicago, Illinois, USA

**Keywords:** HIV-1, CD4^+^T cell subset, CCR5, tropism, pathogenesis

## Abstract

**Background:**

Low HIV-1 infection level in the central memory CD4^+^ T cell subset is a hallmark of both non-progressive HIV infection and non-pathogenetic SIV infection in the natural hosts. However, an important gap in knowledge is whether CCR5-tropic HIV-1 variants have different memory CD4^+^ T cell subset preferences.

**Case Summary:**

Here, we identified clear compartmentalization of two CCR5-tropic HIV-1 in different memory CD4^+^ T cell subsets in a rapid progressor. Participant 40512 was identified in the RV217 cohort. While the transmitted/founder (T/F) virus in 40512 was compartmentalized in the central memory CD4^+^ T cells, the superinfecting virus was compartmentalized in the effector memory CD4^+^ T cells. Both viruses rely on CCR5 to infect primary CD4^+^ T cells. The T/F virus is more than 100-fold more resistant to the CCR5 inhibitor Maraviroc than the superinfecting virus.

**Conclusion:**

This case report demonstrates that CCR5 HIV-1 variants have distinct memory CD4^+^ T cell subset preferences *in vivo*. Because CD4^+^ T cell subset targeting is highly relevant for HIV-1 pathogenesis, understanding the underlying molecular mechanisms may provide deeper insights into HIV-1 therapeutics and functional cure.

## INTRODUCTION

CCR5-tropic HIV-1 mainly infects memory CD4^+^ T cells. Memory CD4^+^ T cells are heterogeneous and comprise central memory (CM), transitional memory (TM), and effector memory (EM) subsets based on cell differentiation stages (1). These memory cell subsets differ especially in their location in the body and the migratory phenotype. Previous studies demonstrate that low HIV-1 viral burden in the CM CD4^+^ T cells correlates with long-term non-progressive HIV-1 infection (2, 3) and is a hallmark of non-pathogenetic SIV infection (4, 5). These findings indicate the importance of CD4^+^ T cell subset tropism of HIV/SIV in determining viral pathogenesis and disease progression. Of note, previous studies including ours showed that CXCR4 tropic HIV-1, which is more virulent than CCR5-tropic virus, preferentially infect the naïve and CM CD4^+^ T cells *in vivo* (6–8). However, an important, yet unanswered question is whether CCR5-tropic HIV-1 variants have different memory CD4^+^ T cell subset preferences, which could determine the rate of disease progression. Here, we demonstrate clear compartmentalization of two CCR5-tropic HIV-1 in different memory CD4^+^ T cell subsets in a rapid progressor.

## CASE PRESENTATION

**Fig 1:**
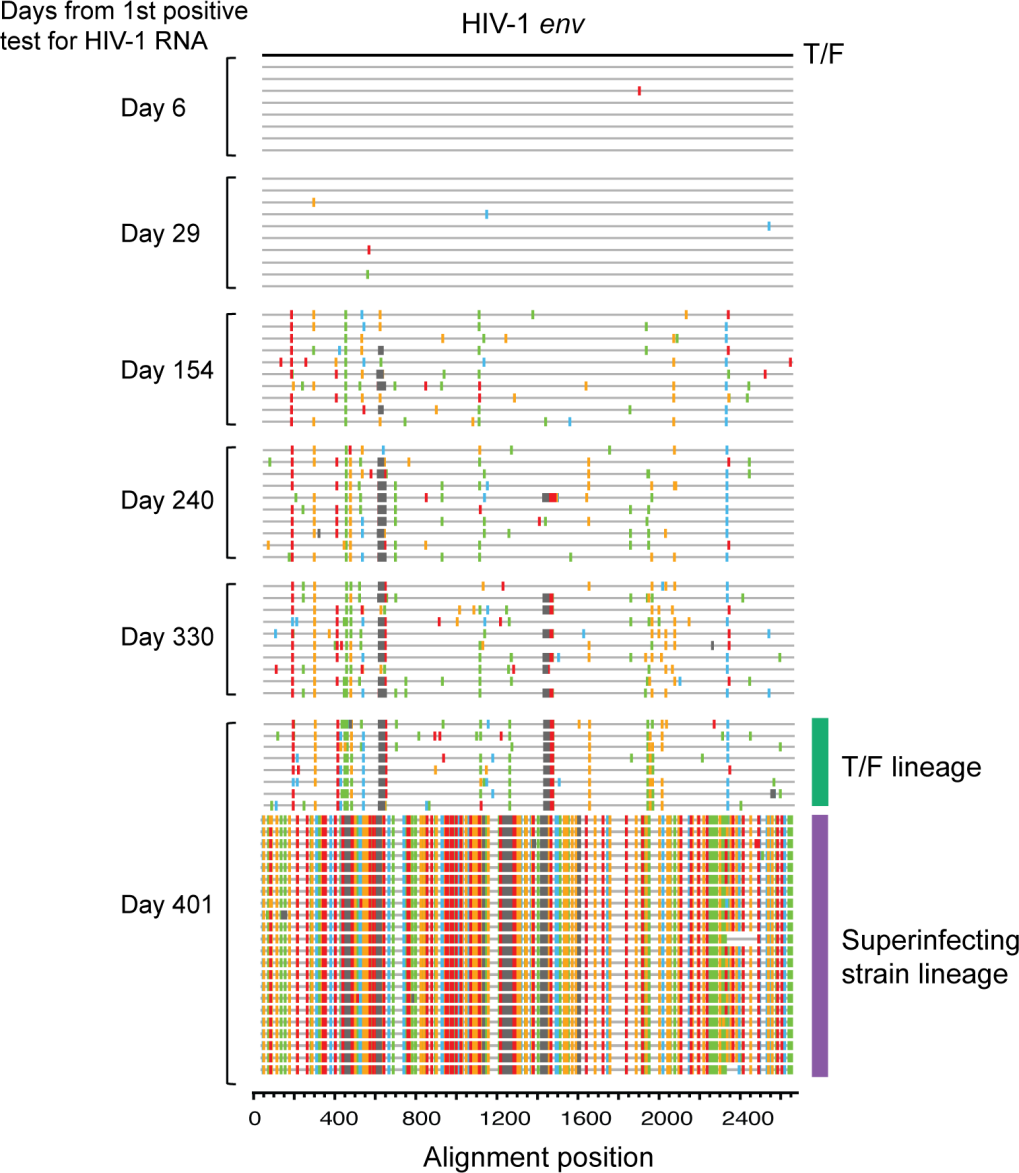
Highlighter plot showing longitudinal virus evolution in participant 40512. The T/F virus (defined as the consensus sequence of day 6) is used as the reference (master) sequence. The superinfecting strain was first detected at day 401. The T/F lineage and the superinfecting lineage at day 401 are color-coded.

Participant 40512 was identified in the RV217 Thailand cohort (9). The RV217 cohort followed at-risk participants twice weekly to identify HIV RNA within a few days of the last HIV negative test, thus allowing investigation of HIV evolution and host immune response from the earliest days of infection (9). Longitudinal virus sequencing demonstrated that participant 40512 was initially infected by a single transmitted/founder (T/F) virus and was superinfected on day 401 (Fig. 1). Both the T/F virus and the superinfecting virus are CRF01_AE.

**Fig 2:**
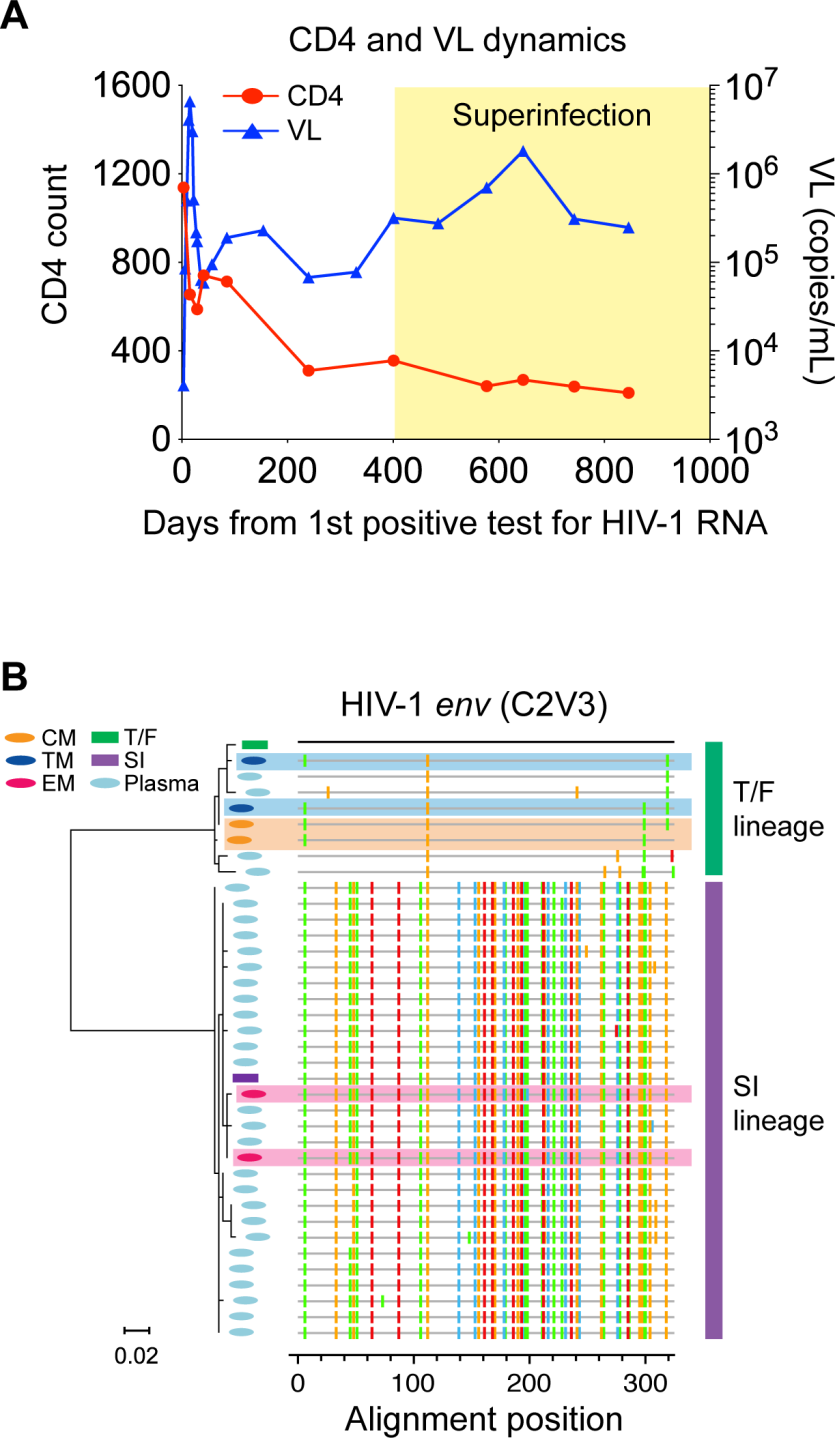
(**A**) CD4 and VL dynamics in 40512. The time frame after superinfection is indicated in yellow. (**B**) Phylogenetic tree and highlighter plot showing the evolutionary relationship between viruses in plasma and in each CD4^+^ T cell subset. Different viruses are color-coded in the tree. Viral sequences in the CM, TM, and EM CD4^+^ T cells are shaded in yellow, blue, and pink, respectively. Viral sequences in each CD^+^ T cell subset were obtained by two independent experiments.

In our previous study on HIV-1 coreceptor switch, we investigated virus coreceptor usage for 21 RV217 Thailand participants at 2 years after HIV-1 transmission, by both virus isolation and deep sequencing (7). While participant 40512 did not harbor CXCR4 virus, this participant had the fastest CD4 decline rate among participants who harbored CCR5 virus alone (0.76 cells/µL/day vs 0.23 cells/µL/day) (7). The viral load (VL) set point in 40512 was relatively high (Fig. 2A) (detailed information on CD4 and VL dynamics for RV217 Thailand participants was reported in our previous studies [6, 7]). Of note, the rapid CD4 loss in 40512 occurred before superinfection (Fig. 2A), suggesting that it was caused by the T/F virus rather than the superinfecting (SI) strain. After superinfection, the superinfecting virus was predominant in plasma, while the original T/F virus became a minor lineage (Fig. 1).

Like other RV217 participants who harbored CCR5 virus alone, participant 40512 had undetectable cell-associated HIV-1 RNA in the naïve CD4^+^ T cells, but cell-associated HIV-1 RNA was detected in all memory CD4^+^ T cell subsets as shown in our previous study (7). Sequencing of the HIV-1 RNA in each memory CD4^+^ T cell subset identified clear compartmentalization of the T/F virus and the superinfecting strain (Fig. 2B). While the T/F lineage was replicating in the CM and TM CD4^+^ T cells, the superinfecting lineage was replicating in the EM CD4^+^ T cells (Fig. 2B). Phylogenetic analysis showed that the superinfecting strain, which was predominant in plasma, originated in the EM CD4^+^ T cells, while the T/F lineage originated in the CM and TM CD4^+^ T cells (Fig. 2B). The compartmentalization was confirmed by two independent cell sorting and HIV-1 RNA sequencing experiments (Fig. 2B). These data demonstrate that the T/F virus and the superinfecting strain preferentially infect different memory CD4^+^ T cell subsets *in vivo*. The EM CD4^+^ T cells, which were preferentially infected by the superinfecting strain, released more virions into the plasma than the CM and TM CD4^+^ T cells. While longitudinal PBMC samples were not available for this study, longitudinal HIV-1 sequencing using plasma samples showed that the superinfecting strain remained predominant in plasma for all subsequent time points (data not shown).

**Fig 3:**
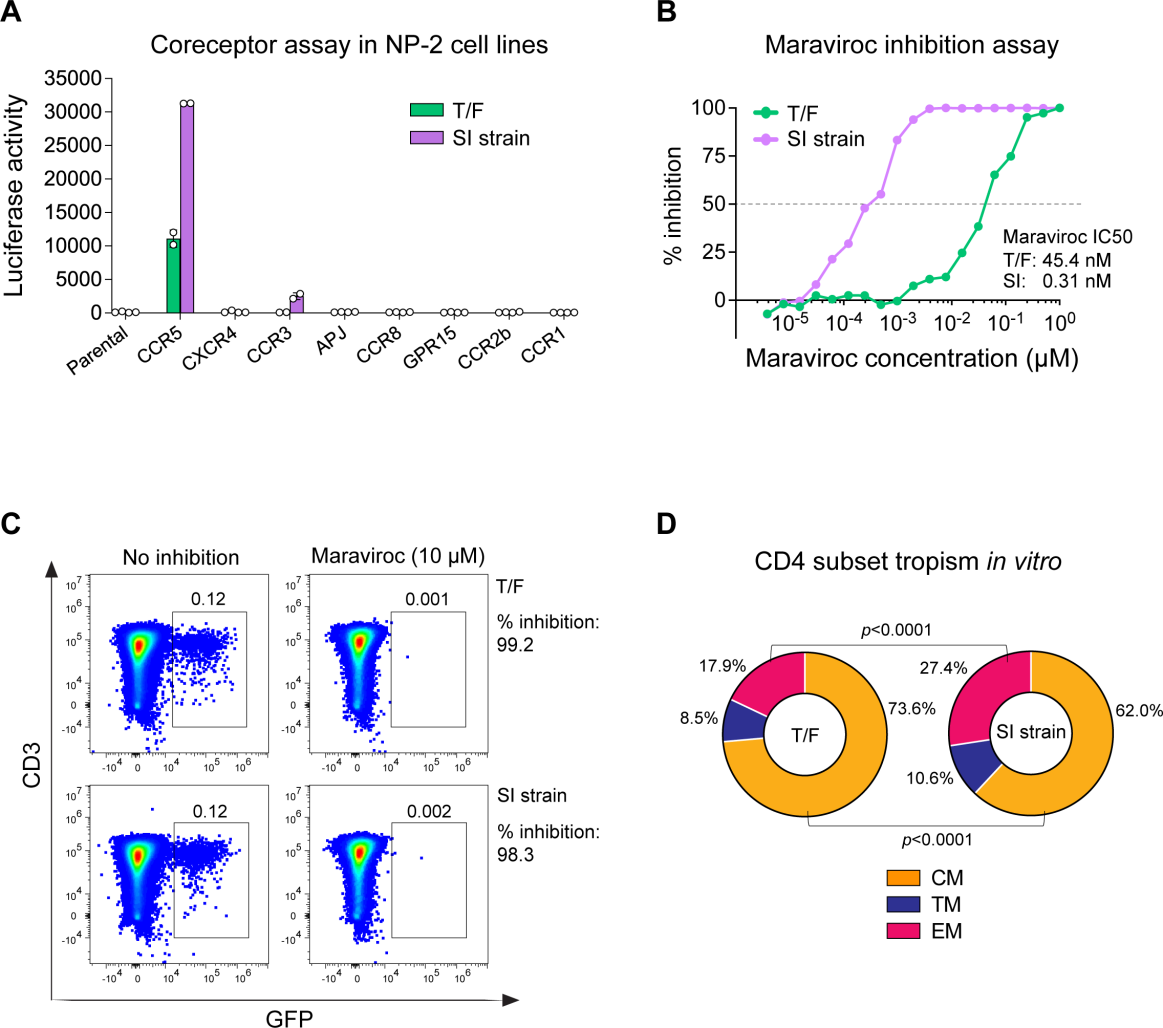
(**A**) Coreceptor usage of the T/F virus and the SI strain in a panel of NP-2 cell lines. The experiments were performed in duplicate, and the error bar shows the standard deviation. (**B**) Virus sensitivity to Maraviroc inhibition in the NP-2 CCR5 cell line. The Maraviroc IC_50_ of each virus is shown. (**C**) Virus sensitivity to Maraviroc inhibition in primary CD4^+^ T cells. The percentage of inhibition for each virus is shown. (**D**) *In vitro* CD4^+^ T cell subset tropism of the T/F virus and the superinfecting virus. The percentage of each CD4^+^ T cell subset among the total infected cells is shown. The statistical significance is determined by a chi-squared test.

Coreceptor assay confirmed that both viruses are CCR5-tropic (Fig. 3A). The superinfecting virus could also use CCR3 with low efficiency (Fig. 3A). In NP-2 CCR5 cells, the infectivity of both viruses can be completely inhibited by 1 µM of the CCR5 inhibitor Maraviroc. However, the T/F virus had a 146-fold higher Maraviroc IC_50_ than the superinfecting strain (45.5 nM vs 0.31 nM) (Fig. 3B). These data suggest that these two viruses might use CCR5 in different ways (e.g., use different CCR5 conformations). In primary CD4^+^ T cells, the infectivity of both viruses can be nearly completely inhibited by 10 µM Maraviroc (Fig. 3C). Therefore, both viruses rely on CCR5 to enter primary CD4^+^ T cells, while the contributions of other coreceptors are very minimal, if at all present.

We next determined whether the two viruses preferentially infect different CD4^+^ T cell subsets *in vitro*. Purified CD4^+^ T cells were infected by pseudoviruses containing the GFP reporter. Among the cells infected by the T/F virus, 73.6% were CM, 8.5% were TM, and 17.9% were EM. Among the cells infected by the SI strain, 62% were CM, 10.6% were TM, and 27.4% were EM (Fig. 3D). This single-round infection assay showed that the T/F virus had an advantage in infecting the CM CD4^+^ T cells over the SI strain (*P* < 0.0001, chi-squared test), while the SI strain had an advantage in infecting the EM CD4^+^ T cells (*P* < 0.0001, chi-squared test). Therefore, their compartmentalization *in vivo* could be determined, at least in part, at the entry level. It is likely that even a small difference in virus entry ability could be amplified after multiple rounds of viral replication *in vivo*, consequently leading to the compartmentalization in different memory CD4^+^ T cell subsets as observed in participant 40512. However, because *in vitro* stimulation may alter the susceptibility of each CD4^+^ T cell subset to HIV-1 infection, the *in vitro* data may not accurately reflect a virus CD4^+^ T cell subset targeting *in vivo*.

## DISCUSSION

The current study shows that CCR5-tropic HIV-1 comprises diverse variants with distinct memory CD4^+^ T cell subset preferences. This finding has implications for better understanding HIV-1 pathogenesis and transmissibility. Multiple lines of evidence suggest that the CD4^+^ T cell subset targeting during HIV and SIV infection could be an important determinant for disease progression. First, low HIV-1 infection burden in the CM CD4^+^ T cells is a hallmark of non-pathogenetic SIV infection in the natural hosts (4, 10, 11). Second, in HIV-1 infection, long-term viremic non-progressors have significantly lower HIV burden in their CM CD4^+^ T cells than progressors (2). Furthermore, CXCR4 tropic HIV-1, which is in general more pathogenic than CCR5 virus, has replication advantage in the CM CD4^+^ T cells *in vivo* in comparison to co-existing CCR5 virus (7). Because the CM CD4^+^ T cells primarily locate in the lymph nodes and are critical for CD4 homeostasis, an important question to address in the future is whether CCR5 variants preferentially targeting the CM CD4^+^ T cells can cause faster disease progression (e.g., the highly virulent subtype B HIV-1 variant recently identified in the Netherlands [12]), and whether long-term viremic non-progressors were infected by viruses with low ability to infect the CM CD4^+^ T cells. Regarding HIV-1 transmissibility, because the EM CD4^+^ T cells are mainly distributed in the mucosal sites where most HIV-1 transmissions occur, whether CCR5 variants preferentially infecting the EM CD4^+^ T cells have higher transmissibility requires further study.

Another important question to address in the future is the molecular mechanisms for the distinct memory CD4^+^ T cell subset tropism of the CCR5 HIV-1. While previous studies indicate that CCR5 molecules exist as different conformational states on the cell surface (13), it remains unknown whether different memory CD4^+^ T cell subsets express CCR5 as different conformational forms, which is responsible for their unequal susceptibility to different CCR5 variants. The *in vitro* experiment using pseudovirus showed different CD4^+^ T cell subset preferences at the entry level. Several reasons might explain why both the T/F and the SI virus could infect all memory CD4^+^ T cell subsets *in vitro*. First, *in vitro* stimulation may alter the phenotype of each cell subset. For example, the CM CD4^+^ T cells, which are at resting state *in vivo*, may become more susceptible to HIV-1 infection after *in vitro* stimulation. Second, a modest difference in virus entry ability, as observed in the single-round infection assay, will be amplified after multiple rounds of viral replication cycles *in vivo*, leading to a clear compartmentalization as observed in participant 40512. This possibility needs to be further determined by virus competition assay using infectious molecular clones. Additionally, potential post-entry mechanisms could also exist. In summary, characterization of the underlying molecular mechanisms is expected to provide deeper insights into HIV-1 prevention, treatment, and functional cure.

## Data Availability

HIV-1 sequences used in the current study were previously deposited in GenBank (MN792521-MN792549; OM826457-OM826666). All data are available upon request through the corresponding author.
